# Effectiveness of antimicrobial agent combinations against carbapenem-producing *Klebsiella pneumoniae* with KPC variants in China

**DOI:** 10.3389/fmicb.2024.1519319

**Published:** 2025-01-22

**Authors:** Congcong Liu, Yuchen Wu, Yanyan Zhang, Zelin Yan, Danxia Gu, Hongwei Zhou, Ning Dong, Chang Cai, Gongxiang Chen, Rong Zhang

**Affiliations:** ^1^Department of Clinical Laboratory, Tianjin First Central Hospital, School of Medicine, Nankai University, Tianjin, China; ^2^Department of Clinical Laboratory, Second Affiliated Hospital of Zhejiang University, School of Medicine, Hangzhou, China; ^3^Department of Clinical Laboratory, Zhejiang Provincial People's Hospital, Hangzhou, China; ^4^MOE Key Laboratory of Geriatric Diseases and Immunology, Suzhou Key Laboratory of Pathogen Bioscience and Anti-infective Medicine, School of Biology & Basic Medical Sciences, Suzhou Medical College, Soochow University, Suzhou, China; ^5^College of Animal Science and Technology, College of Veterinary Medicine, Zhejiang Agricultural and Forestry University, Hangzhou, China

**Keywords:** carbapenem-resistant *Klebsiella pneumoniae*, checkerboard assay, antimicrobial agent combinations, KPC variant, ceftazidime/avibactam-based dual combinations

## Abstract

**Purpose:**

Carbapenem-resistant *Klebsiella pneumoniae* (CRKP) producing carbapenemases poses a global threat to public health. Antimicrobial agent combinations have been promoted as a potential therapeutic strategy for infections. The most effective antimicrobial combinations against CRKP strains producing different carbapenemases are currently unclear, particularly those producing the KPC variant carbapenemases. This study is aimed to evaluate the effectiveness of various antimicrobial agent combinations against CRKP strains with different carbapenemases.

**Methods:**

A checkerboard assay involving 24 antimicrobial agent combinations was conducted on 44 strains of carbapenemase-producing CRKP isolated from patients of which 13 CRKP strains carried single KPC variants. The 24 antimicrobial combinations were based on meropenem, polymyxin, tigecycline, ceftazidime/avibactam, respectively. The fractional inhibitory concentration (FIC) indexes were calculated for each combination of antimicrobial agents.

**Results:**

The distribution of carbapenemases in 44 CRKP strains was as follows: KPC variants (*n* = 13, 29.5%), KPC-2 (*n* = 10, 22.7%), metallo-*β*-lactamases (*n* = 9, 20.5%), OXA-48-like (*n* = 12, 27.3%). In the checkerboard assay, the combination of polymyxin and aztreonam exhibited the highest synergistic effect against CRKP strains, with a rate of 95.5% (42/44). This was followed by polymyxin-meropenem at 88.6% (39/44) and polymyxin-levofloxacin at 68.2% (30/44). Additionally, polymyxin-aztreonam combination and polymyxin-meropenem showed the highest sum of synergistic and additive rates of 100.0% against KPC variant-producing CRKP strains. Notably, ceftazidime/avibactam-based combinations exhibited better synergistic effects on KPC variant-producing CRKP strains compared to other CRKP strains with adjusted *p* value <0.05.

**Conclusion:**

Our study suggests that the combinations of antimicrobial agent could serve as potential treatment strategies against CRKP infections. Furthermore, the effectiveness of these combinations is influenced by the types of carbapenemases present. Ceftazidime/avibactam-based combinations have showed superior synergistic effects on KPC variant-producing CRKP strains.

## 1 Introduction

*Klebsiella pneumoniae* is a common Gram-negative opportunistic pathogen causing gastrointestinal, urinary tract, respiratory and blood-stream infections in clinical practice (Xu et al., 2017; Wyres et al., 2020). Carbapenems are known as ‘last-resort’ *β*-lactam antimicrobial agents for controlling and treating *K. pneumoniae* infections (Queenan and Bush, 2007). However, the detection rates of carbapenem-resistant *K. pneumoniae* (CRKP) have shown an increasing trend annually in recent years (Holt et al., 2015; Wyres et al., 2020). The production of carbapenemases is the primary mechanism mediating carbapenem resistance. Carbapenemases are typically categorized into three major classes: *K. pneumoniae* carbapenemase (KPC) (Ambler class A with serine-based hydrolytic activities), metallo-*β*-lactamases (MβL) (Ambler class B with zinc in their active sites, e.g., NDM), and OXA-48 (Ambler class D). KPC carbapenemase is the most prevalent class worldwide, especially KPC-2 and KPC-3 (Ferrari et al., 2019; Wyres et al., 2020; Li et al., 2022; Zhang et al., 2023).

The high incidence of CPKP infections significantly increases the healthcare costs, as well as the morbidity and mortality rates of patients (Wyres et al., 2020). A prospective, multicenter, cohort study showed that the unadjusted 30-day mortality rate due to CRKP infection stood at 12–28% globally (Wang et al., 2022). Besides, a systematic review and meta-analysis indicated that patients infected with CRKP have higher pooled mortality (42.14%) compared to those with carbapenem-susceptible *K. pneumoniae* (CSKP) infections (21.16%) (Xu et al., 2017). Effectively treating the CRKP infections presents a major challenge to global health. The options for antimicrobial treatment against CRKP infections are limited, with only a few antibiotics are available, including polymyxins, tigecycline, aminoglycosides, fosfomycin and ceftazidime/avibactam (Livermore, 2005; Falagas et al., 2016; Shi et al., 2020; Mohapatra et al., 2021). However, resistance to these agents has been increasingly reported (Gu et al., 2016; Zhang et al., 2017; Lv et al., 2020). Among them, ceftazidime/avibactam, a novel *β*-lactam-plus-β-lactamase inhibitor combination, is known to be effective against serine β-lactamases but not against metallo-β-lactamases (Shi et al., 2020). With the widespread use of ceftazidime/avibactam, more than 150 KPC-2/KPC-3 variants (hereinafter referred to as KPC variants) that confer resistance to ceftazidime/avibactam have been reported in clinics worldwide, with the majority of these variants discovered in the past 3 years (Mueller et al., 2019; Wu et al., 2022). Compared to KPC-2/KPC-3, the new variants have undergone insertions, deletions, and/or point mutations, altering the KPC structure and enhancing the affinity for ceftazidime while weakening the affinity for avibactam (Mueller et al., 2019; Wu et al., 2022). In the face of rising antibiotic resistance, treating bacterial infections are increasingly difficult.

In the current era, characterized by a low success rate in antibiotic drug discovery, combining two or more antimicrobial agents to treat a single infection, referred to as antimicrobial agent combinations, may present a viable alternative for treating CRKP infections. The European Society of Clinical Microbiology and Infectious Diseases (ESCMID) guidelines propose that, for patients with severe infections caused by carbapenem-resistant *Enterobacterales* (CRE) strains that exhibit *in vitro* susceptibility only to polymyxins, aminoglycosides, tigecycline, or fosfomycin, or in the case where *β*-lactam/β-lactamase inhibitor combinations are unavailable, it is advisable to pursue a therapeutic combination regimen active *in vitro* (Paul et al., 2022). Antimicrobial agent combinations can exploit four principal effects on microbes: additive effects, where the impact is the same as the combined effects of the individual antibiotics; synergistic effects, where the impact is greater than the sum of the individual antibiotics’ effects; antagonistic effects, where the impact is reduced compared to the combined effects of the individual antibiotics; and indifferent effects, where one antimicrobial agent can’t affect the activity of the other. Most antimicrobial agent combinations produce a synergistic effect and are more potent than either monotherapy (Torella et al., 2010). Indeed, a noteworthy 30% of the novel antibiotics sanctioned by the FDA during the preceding five-year period have been combination therapies (Geraldine et al., 2020). A systematic review, which analyzed 136 studies, revealed that combinations based on colistin and/or carbapenems demonstrated a notable synergistic effect on CRKP strains. This combination significantly enhanced bactericidal activity while concurrently reducing the emergence of antibiotic-resistant bacteria (Scudeller et al., 2021). Notably, a study found that the effectiveness of different antimicrobial agent combinations was influenced by CRKP infections with different resistance mechanisms (Liu et al., 2021). Wu et al. also reported the ceftazidime/avibactam-meropenem combination was highly synergistic against KPC-producing isolates (91.3%) and carbapenemase-non-producing isolates (100%) (Yun et al., 2024). Moreover, the therapeutic strategy of some antimicrobial combinations can also minimize side effects associated with medication dosage for the patient, as it allows for the use of reduced concentrations of each drug, especially in cases where long-term use of antibiotics causes side effects (Frank et al., 2022).

So far, a large amount of researches on the emergence of carbapenem resistance among *K. pneumoniae* are widely available. Nevertheless, results on the valid antimicrobial agent combinations for the treatment for CRKP infections, particularly for those producing the KPC variant carbapenemases, are limited. In this study, we conducted a comprehensive analysis to explore the *in vitro* efficacy of 24 antimicrobial agent combinations based on meropenem, polymyxin, tigecycline, ceftazidime/avibactam, respectively, against 44 CRKP strains that encoded carbapenemases, especially those producing the KPC variant of carbapenemases.

## 2 Materials and methods

### 2.1 Strain collection

In this study, 44 carbapenemase-producing CRKP strains were collected from patients in China enrolled in this study. 44 CRKP strains were isolated from sputum, feces, blood and abdominal fluid samples. Thirteen CRKP strains carried KPC variants. Ten CRKP strains produced KPC-2 carbapenemase. Nine CRKP strains carried metallo-*β*-lactamases carbapenemases. Twelve CRKP strains produced OXA-48-like carbapenemases. All strains were identified using the matrix-assisted laser desorption/ionization-time-of-flight mass spectrometry (Bruker Daltonik GmbH, Bremen, Germany). Polymerase chain reactions (PCRs) were conducted to detect carbapenemase genes (*bla*_NDM_, *bla*_IMP_, *bla*_KPC_, *bla*_VIM_, and *bla*_OXA-48_), as previously described (Zhang et al., 2017). And then the positive products were confirmed through Sanger sequencing. The whole-genome sequencing (WGS) was performed to identify the sequence types (ST) defined by multilocus sequence typing (MLST) and accurate carbapenemase and KPC allelic variants.

### 2.2 WGS and MLST analysis

The bacterial DNAs were extracted from overnight cultures using the Hipure Bacterial DNA kit (Magen, Shanghai, China). WGS was performed with Illumina (Illumina, San Diego, CA, USA). *De novo* assembly of the raw reads was performed with SPAdes v3.13.1 (Bankevich et al., 2012). Antibiotic resistance genes were identified by ResFinder v4.1.11 (Zankari et al., 2012) and the STs were determined using the SRST2 v0.2.0 (Inouye et al., 2014).

### 2.3 Synergy testing by checkerboard assay

The minimal inhibitory concentrations (MIC) of tigecycline, polymyxin, meropenem, imipenem, ceftazidime/avibactam, aztreonam, fosfomycin, amikacin, levofloxacin, cefoperazone/sulbactam, piperacillin/tazobactam, and cefepime against 44 carbapenemase-producing CRKP strains were determined using broth microdilution and interpreted according to the Clinical and Laboratory Standards Institute (CLSI) guidelines. The *in vitro* synergistic bactericidal activity of combined antibiotics was assessed at various concentrations using checkerboard synergy testing as described previously (Elemam et al., 2010). The 96-well microtiter plates were prepared with increasing concentrations of one drug, ranging from 0.125 to 8 times the MIC, along the x-axis, and increasing concentrations of a second drug, also ranging from 0.125 to 8 times the MIC, along the y-axis. The results of the antimicrobial susceptibility tests were interpreted in accordance with the 2022 CLSI guidelines (CLSI, 2022).

The synergistic effects were evaluated by determining the fractional inhibitory concentration (FIC) indexes. The FIC index was calculated as follows: FIC = (MIC A drug combination/MIC A drug alone) + (MIC B drug combination/MIC B drug alone). The results were interpreted based on the following criteria: FIC ≤ 0.5, synergism, indicating that the combined effect of two drugs compounds is significantly greater than the individual drug effect; 0.5 < FIC ≤1, additive, indicating that the combined effect of two drugs compounds is slightly greater than the individual drug effect; 1 < FIC ≤2, indifferent, indicating that there is no difference between the combined effect of two drugs compounds and the individual drug effect; FIC >2, antagonistic, indicating that the activity of one antimicrobial drug is diminished by another drug.

### 2.4 Data analysis

Statistical analyses were conducted using R software version 4.1.1. For comparisons, Fisher’s exact test was applied, along with the Benjamini and Hochberg correction for multiple comparisons.

## 3 Results

### 3.1 Characteristics of CRKP isolates

This study involved 44 CRKP isolates. Among these, ten strains produced the KPC-2 carbapenemases, and the distribution of ST types was as follows: nine strains belonged to ST11, and the remaining two strains belonged to ST15 and ST3627, respectively. Thirteen strains produced twelve KPC variants, including KPC-14, KPC-25, KPC-33, KPC-35, KPC-51, KPC-52, KPC-71, KPC-76, KPC-77, KPC-78, KPC-93 and KPC-112. The distribution of ST types was as follows: twelve strains belonged to ST11, and the remaining one strain belonged to ST859. Additionally, twelve strains harbored OXA-48-like carbapenemases, and the distribution of ST types was as follows: eleven strains belonged to ST15, and the remaining one strain belonged to ST37. Nine strains were producers of metallo-*β*-lactamases. The distribution of ST types was as follows: four strains belonged to ST15, four strains belonged to ST307, and the remaining one strain belonged to ST380.

### 3.2 The *in vitro* synergism effects using the checkerboard method

The following twenty-four antimicrobial combinations were tested: tigecycline-aztreonam, tigecycline-imipenem, tigecycline-fosfomycin, tigecycline-amikacin, tigecycline-meropenem, tigecycline-levofloxacin, meropenem-cefoperazone/sulbactam, meropenem-piperacillin/tazobactam, meropenem-aztreonam, meropenem-cefepime, meropenem-amikacin, meropenem-levofloxacin, polymyxin-fosfomycin, polymyxin-amikacin, polymyxin-meropenem, polymyxin-levofloxacin, polymyxin-aztreonam, polymyxin-imipenem, ceftazidime/avibactam-fosfomycin, ceftazidime/avibactam-amikacin, ceftazidime/avibactam-aztreonam, ceftazidime/avibactam-imipenem, ceftazidime/avibactam-meropenem and ceftazidime/avibactam-levofloxacin.

Although the synergistic effect and the additive effect differ in drug combination therapy, they both represent potential positive outcomes that can arise from the combined use of drugs. In this study, most combinations showed no synergistic effect on the strains, so we added the synergistic and additive effects together before conducting statistical analysis. Unless stated otherwise, the subsequent use of the term “synergistic effect” refers to the combined outcome of both synergistic and additive effects.

**Figure 1:**
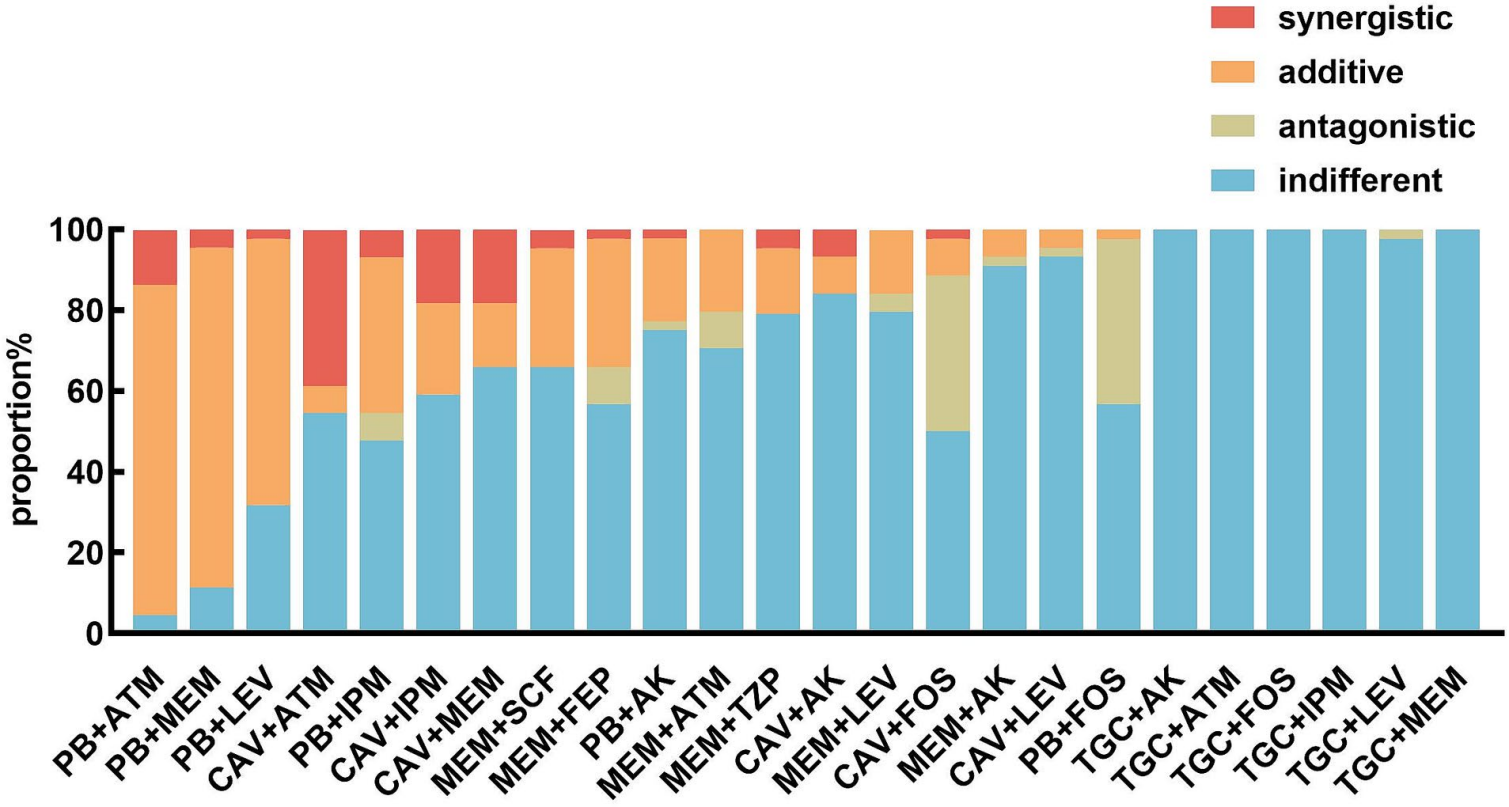
*In vitro* effects of different antimicrobial agent combinations against CRKP strains. PB, polymyxin; TGC, tigecycline; ATM, aztreonam; IPM, imipenem; FOS, fosfomycin; AK, amikacin; MEM, meropenem; LEV, levofloxacin; SCF, cefoperazone/sulbactam; TZP, piperacillin/tazobactam; FEP, cefepime; CAV, ceftazidime/avibactam.

**Figure 2:**
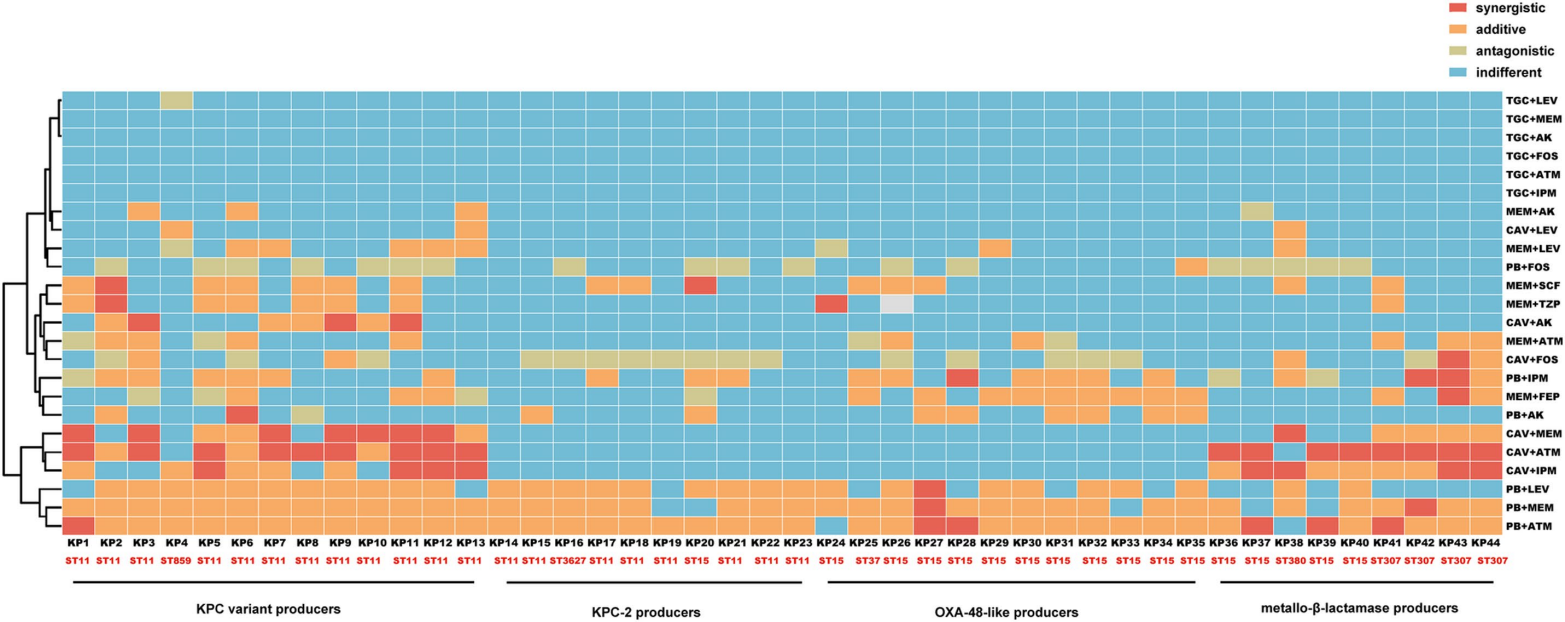
Heatmap of *in vitro* effects of different antimicrobial agent combinations against CRKP strains. The vertical and horizontal axes represent antimicrobial agent combinations and CRKP strains, respectively.

The polymyxin-aztreonam combination exhibited the highest synergistic effect at 95.5% (42/44), followed by polymyxin-meropenem at 88.6% (39/44), and polymyxin-levofloxacin at 68.2% (30/44). The synergistic effects of ceftazidime/avibactam-aztreonam at 45.5% (20/44), polymyxin-imipenem at 45.5% (20/44), ceftazidime/avibactam-imipenem at 40.9% (18/44), ceftazidime/avibactam-meropenem at 34.1% (15/44), meropenem-cefoperazone/sulbactam at 34.1% (15/44), and meropenem-cefepime at 34.1% (15/44). Other combinations, including polymyxin-amikacin, polymyxin-fosfomycin, meropenem-aztreonam, meropenem-piperacillin/tazobactam, meropenem-amikacin, meropenem-levofloxacin, ceftazidime/avibactam-amikacin, ceftazidime/avibactam-fosfomycin, ceftazidime/avibactam-levofloxacin, showed synergistic effects ranging from 2.2–21.7%. The tigecycline-based combinations, such as tigecycline-aztreonam, tigecycline-imipenem, tigecycline-fosfomycin, tigecycline-amikacin, tigecycline-meropenem, and tigecycline-levofloxacin, demonstrated indifferent effects on these CRKP isolates (Figures 1, 2).

### 3.3 Evaluation of antimicrobial combinations against CRKP strains with different carbapenemases

**Figure 3:**
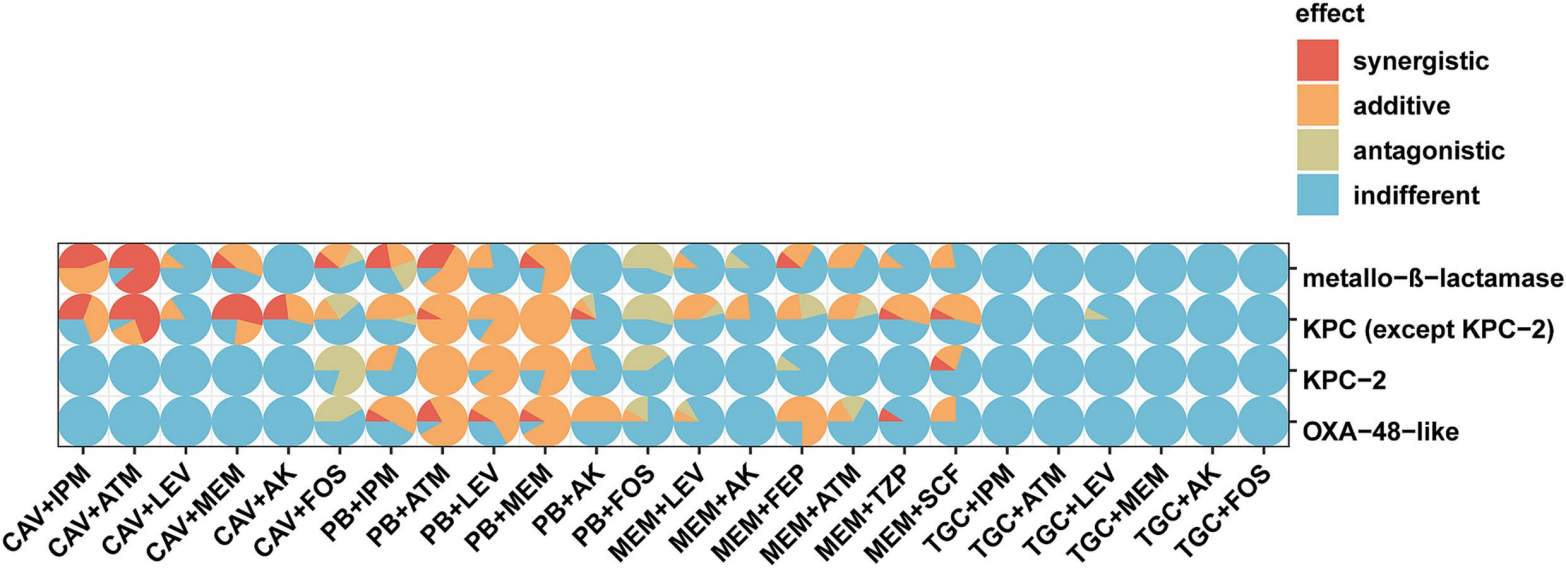
Scatter pie chart of in vitro effects of different antimicrobial agent combinations against CRKP strains. The vertical and horizontal axes represent carbapenemase types and antimicrobial agent combinations, respectively.

The two most effective antimicrobial combinations, polymyxin-aztreonam and polymyxin-meropenem, exhibited no statistically significant differences in their effects against CRKP strains with varying carbapenemases (Figure 1). The polymyxin-aztreonam combination showed the highest synergistic effect against KPC-2-producing CRKP strains at 100.0%, followed by polymyxin-levofloxacin at 90.0% and polymyxin-meropenem at 80.0%. Notably, the efficacy of the polymyxin-levofloxacin combination against KPC-2-producing CRKP strains was significantly higher than its efficacy against metallo-*β*-lactamase-producing CRKP strains (*p* < 0.05) (Figure 3).

In the study, the ceftazidime/avibactam-imipenem combination demonstrated the highest synergistic rate of 100.0% against metallo-β-lactamase-producing isolates. This was closely followed by polymyxin-aztreonam (88.9%), ceftazidime/avibactam-aztreonam (88.9%), polymyxin-meropenem (77.8%), and ceftazidime/avibactam-meropenem (55.6%) (Figure 3). The ceftazidime/avibactam-aztreonam, ceftazidime/avibactam-imipenem and ceftazidime/avibactam-meropenem combinations showed greater activity against metallo-β-lactamase producers than KPC-2 and OXA-48 producers with adjusted *p* values <0.05.

Against OXA-48-like-producing CRKP strains, polymyxin-aztreonam and polymyxin-meropenem combinations showed the highest synergistic rate at 91.7%. This was followed by the meropenem-cefepime combination (75.0%), polymyxin-levofloxacin (66.7%), polymyxin-imipenem (58.3%) and polymyxin-amikacin (50.0%) (Figure 3). Notably, the efficacy of the meropenem-cefepime combination against OXA-48-like-producing CRKP strains was significantly higher than its effectiveness against KPC-2- producing CRKP strains (*p* < 0.05).

### 3.4 Evaluation of antimicrobial combinations against CRKP strains with KPC variants

Against isolates producing KPC variants, the combinations of polymyxin-aztreonam and polymyxin-meropenem showed the highest synergistic rates at 100.0%. These were followed by ceftazidime/avibactam-aztreonam at 92.3%, polymyxin-levofloxacin at 84.6%, ceftazidime/avibactam-meropenem at 76.9%, ceftazidime/avibactam-imipenem at 69.2%, meropenem-cefoperazone/sulbactam, meropenem-piperacillin/tazobactam and ceftazidime/avibactam-amikacin each at 53.8% (Figure 3). Notably, the synergistic effects of the ceftazidime/avibactam-aztreonam, ceftazidime/avibactam-meropenem, ceftazidime/avibactam-imipenem and ceftazidime/avibactam-amikacin combinations against KPC variant-producing CRKP strains were significantly higher than their effects against KPC-2-producing CRKP strains (Table 1) and OXA-48-like-producing CRKP strains. The adjusted *p* values for these comparisons were all below 0.05. The efficacy of ceftazidime/avibactam-amikacin combination against KPC variant-producing CRKP strains was significantly higher than its effectiveness against metallo-*β*-lactamase-producing CRKP strains (*p* < 0.05).

**Table 1:** In vitro combination effects of different regimens against CRKP with KPC variants or KPC-2.

Drug combination	KPC variants (except KPC-2)				KPC-2			
	Synergistic	Additive	Antagonistic	Indifferent	Synergistic	Additive	Antagonistic	Indifferent
CAV + AK	3 (23.1)	4 (30.8)	0 (0.0)	6 (46.2)	0 (0.0)	0 (0.0)	0 (0.0)	10 (100.0)
CAV + ATM	9 (69.2)	3 (23.1)	0 (0.0)	1 (7.7)	0 (0.0)	0 (0.0)	0 (0.0)	10 (100.0)
CAV + FOS	0 (0.0)	2 (15.4)	3 (23.1)	8 (61.5)	0 (0.0)	0 (0.0)	8 (80.0)	2 (20.0)
CAV + IPM	4 (30.8)	5 (38.5)	0 (0.0)	4 (30.8)	0 (0.0)	0 (0.0)	0 (0.0)	10 (100.0)
CAV + LEV	0 (0.0)	2 (15.4)	0 (0.0)	11 (84.6)	0 (0.0)	0 (0.0)	0 (0.0)	10 (100.0)
CAV + MEM	7 (53.8)	3 (23.1)	0 (0.0)	3 (23.1)	0 (0.0)	0 (0.0)	0 (0.0)	10 (100.0)
MEM + AK	0 (0.0)	3 (23.1)	0 (0.0)	10 (76.9)	0 (0.0)	0 (0.0)	0 (0.0)	10 (100.0)
MEM + ATM	0 (0.0)	4 (30.8)	2 (15.4)	7 (53.8)	0 (0.0)	0 (0.0)	0 (0.0)	10 (100.0)
MEM + FEP	0 (0.0)	3 (23.1)	3 (23.1)	7 (53.8)	0 (0.0)	0 (0.0)	1 (10.0)	9 (90.0)
MEM + LEV	0 (0.0)	5 (38.5)	1 (7.7)	7 (53.8)	0 (0.0)	0 (0.0)	0 (0.0)	10 (100.0)
MEM + SCF	1 (7.7)	6 (46.2)	0 (0.0)	6 (46.2)	1 (10.0)	2 (20.0)	0 (0.0)	7 (70.0)
MEM + TZP	1 (7.7)	6 (46.2)	0 (0.0)	6 (46.2)	0 (0.0)	0 (0.0)	0 (0.0)	10 (100.0)
PB + AK	1 (7.7)	1 (7.7)	1 (7.7)	10 (76.9)	0 (0.0)	2 (20.0)	0 (0.0)	8 (80.0)
PB + ATM	1 (7.7)	12 (92.3)	0 (0.0)	0 (0.0)	0 (0.0)	10 (100.0)	0 (0.0)	0 (0.0)
PB + FOS	0 (0.0)	0 (0.0)	7 (53.8)	6 (46.2)	0 (0.0)	0 (0.0)	4 (40.0)	6 (60.0)
PB + IPM	0 (0.0)	6 (46.2)	1 (7.7)	6 (46.2)	0 (0.0)	3 (30.0)	0 (0.0)	7 (70.0)
PB + LEV	0 (0.0)	11 (84.6)	0 (0.0)	2 (15.4)	0 (0.0)	9 (90.0)	0 (0.0)	1 (10.0)
PB + MEM	0 (0.0)	13 (100)	0 (0.0)	0 (0.0)	0 (0.0)	8 (80.0)	0 (0.0)	2 (20.0)
TGC + AK	0 (0.0)	0 (0.0)	0 (0.0)	13 (100.0)	0 (0.0)	0 (0.0)	0 (0.0)	10 (100.0)
TGC + ATM	0 (0.0)	0 (0.0)	0 (0.0)	13 (100.0)	0 (0.0)	0 (0.0)	0 (0.0)	10 (100.0)
TGC + FOS	0 (0.0)	0 (0.0)	0 (0.0)	13 (100.0)	0 (0.0)	0 (0.0)	0 (0.0)	10 (100.0)
TGC + IPM	0 (0.0)	0 (0.0)	0 (0.0)	13 (100.0)	0 (0.0)	0 (0.0)	0 (0.0)	10 (100.0)
TGC + LEV	0 (0.0)	0 (0.0)	1 (7.7)	12 (92.3)	0 (0.0)	0 (0.0)	0 (0.0)	10 (100.0)
TGC + MEM	0 (0.0)	0 (0.0)	0 (0.0)	13 (100.0)	0 (0.0)	0 (0.0)	0 (0.0)	10 (100.0)

## 4 Discussion

The increasing spread of multidrug-resistant (MDR) CRKP strains has further limited therapeutic options for nosocomial infections, resulting in high fatality rates among patients with compromised immunity (Holt et al., 2015). The current therapeutic challenges in treating CRKP infections highlight the urgent need for effective antimicrobial combinations. While numerous studies have explored various antimicrobial agent combinations against CRKP, there is a lack of comprehensive research on therapeutic strategies for CRKPs that produce different carbapenemases, particularly those producing KPC variants. In this study, we identified the *in vitro* efficacy of 24 antimicrobial combinations with different modes of action in treating of 44 carbapenemase-producing CRKP strains. The combination therapy of antimicrobial agents with different mechanisms of action and pharmacokinetic properties can enhance antibacterial efficacy and reduce the emergence of antibiotic resistance.

In this study, the three most effective antimicrobial combinations against CRKP strains were all based on polymyxin, including polymyxin-aztreonam, polymyxin-meropenem and polymyxin-levofloxacin. Polymyxin, a cationic polypeptide, binds to negatively charged lipopolysaccharides (LPS) and disrupts the bacteria’s outer membrane, thereby exerting its antibacterial effect (Mohapatra et al., 2021). Aztreonam and meropenem function by inhibiting the biosynthesis of bacteria cell walls (Hellinger and Brewer, 1999). Levofloxacin, on the other hand, blocks DNA replication, leading to bacterial cell death (Xue et al., 2021). Consequently, these combinations have demonstrated remarkable therapeutic efficacy against CRKP strains through distinct mechanisms. Additionally, the studies by Yu et al. have also observed synergistic effects of polymyxin-meropenem and polymyxin-levofloxacin against polymyxin-resistant CRKP isolates (Yu et al., 2019).

Reports indicate that polymyxin resistance can develop within 24 h of polymyxin monotherapy, but this resistance is mitigated and delayed when combination therapy is used (Le Minh et al., 2015). Significantly, a retrospective cohort study demonstrated that combination therapy with polymyxin-meropenem improved survival rates in patients suffering from bacteremia caused by KPC-producing *K. pneumoniae*, in comparison to patients who received polymyxin monotherapy (Le Minh et al., 2015).

KPC-2 is the most prevalent carbapenemase among CRKP strains in China (Zhang et al., 2017). Ceftazidime/avibactam stands as one of the few effective treatment alternatives against KPC-2-producing CRKP strains. But the clinical use of ceftazidime/avibactam has led to mutations in KPC-2 carbapenemases, emerging as a major mechanisms behind ceftazidime/avibactam resistance (Shi et al., 2020). To date, limited studies have investigated antimicrobial combinations targeting KPC variant-producing CRKP strains. In our study, ten antimicrobial agent combinations exhibited synergistic or addictive effects on ≥50% of KPC variant-producing CRKP strains, while only three combinations showed addictive effects on ≥50% of KPC-2-producing CRKP strains.

In addition to the three antimicrobial agent combinations of polymyxin-aztreonam, polymyxin-meropenem, and polymyxin-levofloxacin, which are beneficial for treating KPC variant-producing and KPC-2-producing CRKP strains, the combination of ceftazidime/avibactam with one other active drug (such as aztreonam, meropenem, imipenem, or amikacin) is also noteworthy. Although the clinical use of ceftazidime/avibactam has contributed to the development of KPC variants and the rapid progression of ceftazidime/avibactam resistance, combining ceftazidime/avibactam with another antimicrobial agent, for example, a carbapenem, may present an alternative therapeutic strategy for ceftazidime/avibactam-resistant KPC variant-producing CRKP strains. Most isolates were resistant to individual antibiotics used in combination. There were some isolates sensitive to both antibiotics used in some antimicrobial agent combinations. In these cases, these combinations exhibited indifferent effects on CRKP strains. But it should be noted that polymyxin-meropenem exhibited additive effects on 7 CRKP strains with KPC variants which were sensitive to polymyxin and meropenem, hinting that antimicrobial agent combinations can serve a crucial function in infections caused by CRKP strains with KPC variants and more effective combination therapy should be explore.

Metallo-*β*-lactamases constitute another common mechanisms for carbapenem resistance in CRKP strains (Zhang et al., 2017). The combination of ceftazidime/avibactam with aztreonam is a promising treatment option for metallo-β-lactamase-producing CRKP strains (Marshall et al., 2017). Previous studies have shown that this combination’s therapeutic effectiveness against bloodstream infections caused by metallo-β-lactamase-producing CRKP strains is superior to other active antibiotics, particularly regarding 30-day all-cause mortality (Falcone et al., 2021).

Our data showed that synergistic activity of the ceftazidime/avibactam plus aztreonam combination was observed in eight out of nine metallo-β-lactamase-producing CRKP strains (88.9%). These eight strains exhibited high resistance to aztreonam, with MIC values ranging from 32 to 256 μg/mL. The remaining NDM-5-producing CRKP strain showed indifferent activity with the ceftazidime/avibactam plus aztreonam combination, due to its susceptibility to aztreonam. This suggests that combining ceftazidime/avibactam is particularly effective in treating infections caused by metallo-β-lactamase-producing CRKP strains with high MICs for both ceftazidime/avibactam and aztreonam.

OXA-48-like enzymes are identified as the third most prevalent carbapenemases among CRKP isolates. Polymyxin-based combination antimicrobial regimens demonstrated comparatively high synergy rates against OXA-48-like-producing CRKP strains. These results are consistent with findings from Balkan’s study, which indicates that polymyxin-based dual combinations lead to significantly better treatment outcomes compared to non-polymyxin based combinations in patients with bloodstream infections caused by OXA-48-like carbapenemase-producing *Enterobacteriaceae* (Balkan et al., 2014). Furthermore, the combination of meropenem and cefepime combination exhibited more favorable effects on OXA-48-like-producing CRKP strains than on KPC-2-producing CRKP strains, potentially due to the difference in affinity for meropenem and cefepime between KPC-2 enzymes and OXA-48-like enzymes. This highlights the need for further in-depth studies on the mechanisms underlying these synergistic effects.

However, there are some limitations of this study. Firstly, the number of CRKP isolates analyzed were relatively small due to the difficulty in obtaining KPC variant-producing CRKP strains. More samples should be included in our future research endeavors. Additionally, it remains uncertain whether the antimicrobial agent combinations that demonstrate good synergistic or addictive effects *in vitro* will also have effective anti-infection impacts *in vivo*. The *in vivo* effects and mechanisms need to be further explored in future studies. Thirdly, the effectiveness of more complicated combinations (like polymyxin, aztreonam and meropenem) aren’t explored. Future research should address this aspect to provide new strategies for treatment of infectious diseases.

In summary, this study has investigated highly effective antimicrobial agent combinations against CRKP isolates producing various carbapenemases. The combinations of polymyxin-aztreonam, polymyxin-meropenem and polymyxin-levofloxacin demonstrated superior synergistic effects and could serve as effective therapeutic options for carbapenemase-producing CRKP strains. Additionally, ceftazidime/avibactam-based dual combinations show promise as reliable treatment regimens against KPC variant-producing CRKP strains. To ensure the viability of these combinations in clinical settings, further mechanistic studies and more extensive clinical trials are urgently needed.

## Data Availability

The original contributions presented in the study are included in the article/supplementary material, further inquiries can be directed to the corresponding author.
